# Supplementary material for: Optimization of Biotinylated RNA or DNA Pull-Down Assays for Detection of Binding Proteins: Examples of IRP1, IRP2, HuR, AUF1, and Nrf2
Source: Int J Mol Sci. 2023 Feb 10;24(4):3604. doi: 10.3390/ijms24043604 (PMC9965622; doi:10.3390/ijms24043604)
Supplement: Supplementary file 1 [file ijms-24-03604-s001.zip › ijms-2136525-supplementary.pptx]

## Slide 1
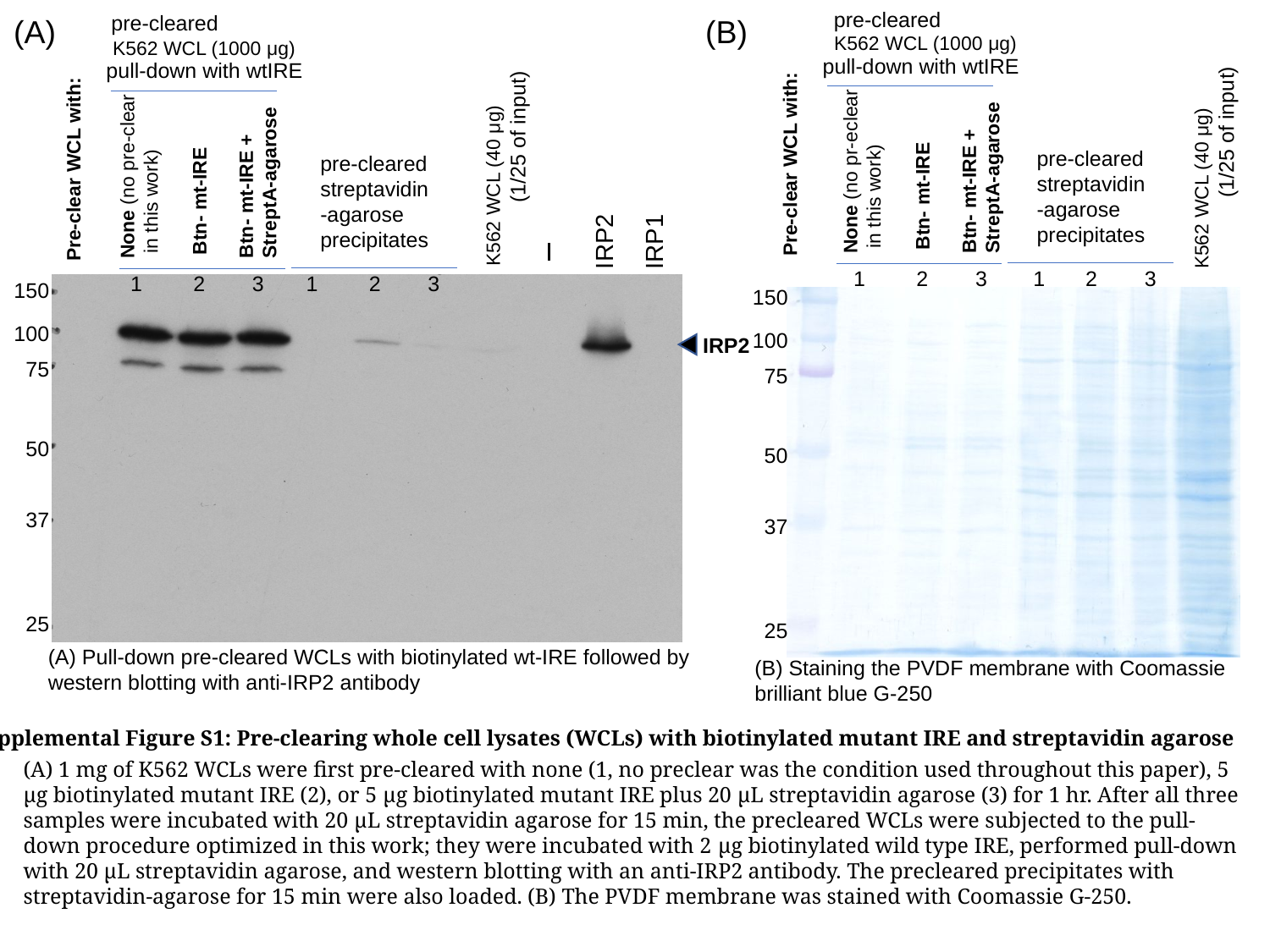

pre-cleared
pre-cleared
(A)
(B)
K562 WCL (1000 μg)
K562 WCL (1000 μg)
pull-down with wtIRE
pull-down with wtIRE
(1/25 of input)
(1/25 of input)
Pre-clear WCL with:
Pre-clear WCL with:
pre-cleared streptavidin-agarose precipitates
None (no pr-eclear in this work)
pre-cleared streptavidin-agarose precipitates
None (no pre-clear in this work)
Btn- mt-IRE + StreptA-agarose
Btn- mt-IRE + StreptA-agarose
K562 WCL (40 μg)
Btn- mt-IRE
K562 WCL (40 μg)
Btn- mt-IRE
IRP2
IRP1
1
2
3
1
2
3
1
2
3
1
2
3
150
150
100
100
IRP2
75
75
50
50
37
37
25
25
(A) Pull-down pre-cleared WCLs with biotinylated wt-IRE followed by western blotting with anti-IRP2 antibody
(B) Staining the PVDF membrane with Coomassie brilliant blue G-250
Supplemental Figure S1: Pre-clearing whole cell lysates (WCLs) with biotinylated mutant IRE and streptavidin agarose
(A) 1 mg of K562 WCLs were first pre-cleared with none (1, no preclear was the condition used throughout this paper), 5 μg biotinylated mutant IRE (2), or 5 μg biotinylated mutant IRE plus 20 μL streptavidin agarose (3) for 1 hr. After all three samples were incubated with 20 μL streptavidin agarose for 15 min, the precleared WCLs were subjected to the pull-down procedure optimized in this work; they were incubated with 2 μg biotinylated wild type IRE, performed pull-down with 20 μL streptavidin agarose, and western blotting with an anti-IRP2 antibody. The precleared precipitates with streptavidin-agarose for 15 min were also loaded. (B) The PVDF membrane was stained with Coomassie G-250.

## Slide 2
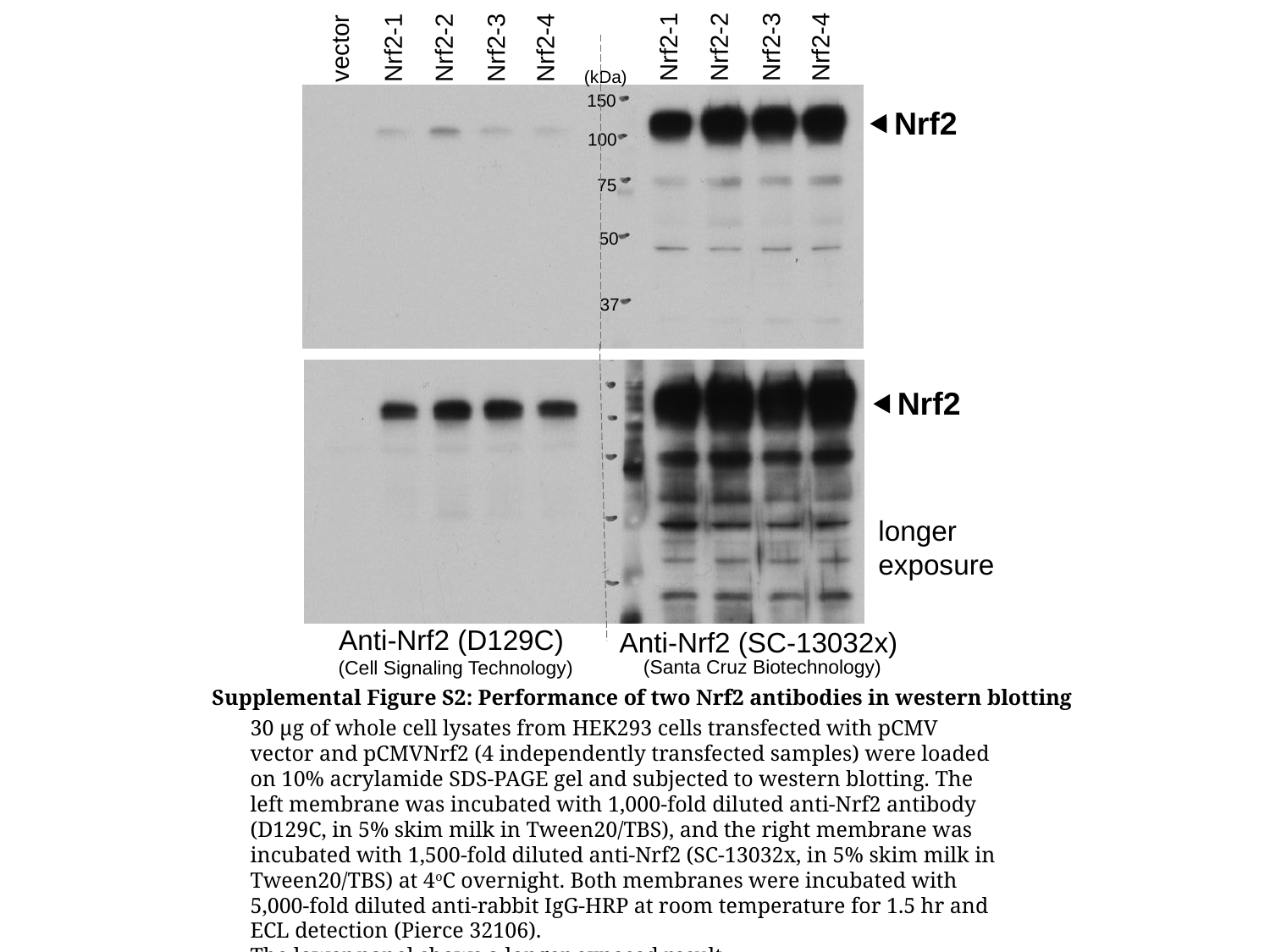

Nrf2-1
Nrf2-2
Nrf2-3
Nrf2-4
Nrf2-1
Nrf2-2
Nrf2-3
Nrf2-4
vector
(kDa)
150
Nrf2
100
75
50
37
Nrf2
longer exposure
Anti-Nrf2 (D129C)
Anti-Nrf2 (SC-13032x)
(Santa Cruz Biotechnology)
(Cell Signaling Technology)
Supplemental Figure S2: Performance of two Nrf2 antibodies in western blotting
30 μg of whole cell lysates from HEK293 cells transfected with pCMV vector and pCMVNrf2 (4 independently transfected samples) were loaded on 10% acrylamide SDS-PAGE gel and subjected to western blotting. The left membrane was incubated with 1,000-fold diluted anti-Nrf2 antibody (D129C, in 5% skim milk in Tween20/TBS), and the right membrane was incubated with 1,500-fold diluted anti-Nrf2 (SC-13032x, in 5% skim milk in Tween20/TBS) at 4oC overnight. Both membranes were incubated with 5,000-fold diluted anti-rabbit IgG-HRP at room temperature for 1.5 hr and ECL detection (Pierce 32106).
The lower panel shows a longer exposed result.
